# Identification and characterization of *Salmonella* spp. from samples of broiler farms in selected districts of Bangladesh

**DOI:** 10.14202/vetworld.2020.275-283

**Published:** 2020-02-13

**Authors:** Debashish Mridha, Md. Nasir Uddin, Badrul Alam, A. H. M. Taslima Akhter, SK. Shaheenur Islam, Md. Saiful Islam, Md. Shahidur Rahman Khan, S. M. Lutful Kabir

**Affiliations:** 1 Department of Microbiology and Hygiene, Bangladesh Agricultural University, Mymensingh 2202, Bangladesh; 2 Food Safety Program, Food and Agricultural Organization, Institute of Public Health, Mohakhali, Dhaka 1215, Bangladesh; 3 Epidemiology Unit, Department of Livestock Services, Krishi Khamar Sarak, Farmgate, Dhaka 1215, Bangladesh

**Keywords:** good agriculture practices, hygienic practices, multidrug resistance, poultry, *Salmonella* spp

## Abstract

**Background and Aim:**

*Salmonella* spp. are an important group of pathogens responsible for human and animal diseases. This study aimed to estimate the prevalence and identify and characterize of *Salmonella* spp. isolated from broiler farms of Gazipur, Tangail, and Dhaka districts of Bangladesh. This study also evaluated the difference of *Salmonella* positivity status between two groups of farms, good practices adapted in broiler rearing at the project intervened farms, and non-project intervened traditional farms.

**Materials and Methods:**

A total of 352 samples including 128 cloacal swabs, 32 whole carcasses, 64 feed, 64 water, and 64 attendants’ hand rinses were collected through convenient sampling technique from 16 poultry food safety project of Food and Agricultural Organization of United Nations Bangladesh intervened farms and other 16 non-project intervened farms in the same location. Various cultural based techniques and biochemical methods were employed for the estimation of prevalence, isolation, and identification of *Salmonella* spp. which was further evaluated by polymerase chain reaction. Antimicrobial susceptibility test using disk diffusion methods and serogrouping by slide agglutination test was accomplished for additional characterization.

**Results:**

Among the samples, an overall prevalence of *Salmonella* spp. was 31.25% (110/352) (95% confidence interval [CI]=26.44-36.38%). However, the prevalence of *Salmonella* spp. was 24.43% (43/176) (95% CI=18.28-31.47) in project intervened farms and 38.07% (67/176) (95% CI=30.87-45.68%) in non-intervened farms. Among the 110 isolates, 31.82% (35/110) were fitted under serogroup B, and the rest of the isolates 75 (68.18%) under serogroup D. Of 110 isolates, 82.72%, 77.27%, 81.82%, and 79.09% were susceptible to ciprofloxacin, gentamycin, norfloxacin, and streptomycin, respectively. In addition, 81.82% and 80% isolates were resistant to erythromycin and tetracycline, respectively. Isolated *Salmonella* spp. presented moderate resistance to both amoxicillin and azithromycin. Alarmingly, 80.91% (89/110) isolates were shown to be multidrug-resistant *Salmonella* spp.

**Conclusion:**

The study has presented a significant variation of the prevalence of *Salmonella* spp. between project intervened and non-project intervened farms, and this indicates project intervened farms are comparatively safer than the non-intervened farms considering public health and food safety grounds. This research outcome also has highlighted a substantial proportion of poultry origin multidrug resistance *Salmonella* spp. is a potential source of public health hazards. In this regard, proper awareness creation and motivational activities on good agriculture practices in poultry rearing and maintaining good personal hygiene at the farmers’ level are warranted through participatory training.

## Introduction

*Salmonella* spp. are commonly responsible for various pathogenic processes in human and animal, including poultry [1]. Among the foodborne diseases caused by bacterial pathogens, *Salmonella* is one of the most important zoonotic pathogens which have more than 2600 serotypes can prompt of human and animal gastrointestinal infection such as gastroenteritis, typhoid fever, paratyphoid fever, and can cause of serious ailments for younger and aged people, and even result of death [2-4]. Human consumed different types of food such as food-producing animals including poultry especially broiler and layer chicken meat, eggs, seafood, beef, pork, vegetables, and contaminated water are the main source of foodborne illness in human [5,6]. It causes endemic salmonellosis worldwide and reasons a colossal economic loss in livestock and poultry industry in Bangladesh [7]. Among the bacterial diseases, *Salmonella* infection is one of the major problems for poultry farming in Bangladesh, which is considered a key threat of the poultry industry [8]. In Bangladesh, the occurrence of *Salmonella* infection is about 21-30% in layer and about 15% in broiler which is measured as the highest prevalence among different types of poultry disease [9,10], among which a variety of acute and chronic diseases in poultry are included [11]. Chicks can be infected with *Salmonella* spp. by vertical transmission through infected parents or by horizontal transmission through hatcheries, sexing in contaminated hatcheries, cloacal infection, and transportation of equipment and feed [12]. Motile *Salmonella* (paratyphoid group) infection causes salmonellosis in chickens with zoonotic significance [13].

It is very common of broiler farming with low or no biosecurity practices in Bangladesh where most of the broiler farms have been developed near the dwellings or close proximate to the human habitats is a significant hazard for public health at present time [14]. In addition, poultry feces are used in the agricultural field and/or as fish feed without proper treatment is deemed to be potentially risky practices for the public health view point. Showing antimicrobials’ resistance by pathogenic bacteria is a universal public health concern throughout the world especially in developing countries [3,14]. The results of imprudent use of antimicrobial agents to minimize bacterial infection or as a growth promoter in poultry production are the major determinants for the emergence of multidrug-resistant pathogenic bacteria [3,15]. Because of the phenomenon of developing multidrug-resistant *Salmonella* isolates, the management of *Salmonella* infection using regular drugs is very difficult [16]. Considering the urgency of the above, the survey of *Salmonella* in food animal production together with surveillance on antimicrobial resistance pattern was very essential [17].

Many previous *Salmonella* studies in country and abroad have used poultry, poultry products and environmental samples for isolation and identification of the organism [7,18-22]. Since, lack of study to evaluating the *Salmonella* spp. from broiler farms with the comparison between two groups of the farm, namely, project-intervened farms with best practices versus non-intervened farm with traditional practices. The farmers of the project-intervened farms were trained on poultry farming in compliance with good practices of biosecurity measures such as provision of perimeter fencing, netting of the farm, footwear clean entry in the farm, all in all-out, and cleaning and sanitation, and judicial use antibiotics through the Food and Agricultural Organization (FAO)-Food Safety Program project intervention to be appropriate for safer poultry production considering public health hazard.

This study aimed to estimate the prevalence of and identify and characterize *Salmonella* spp. isolated from broiler farms of Gazipur, Tangail, and Dhaka districts of Bangladesh. This study also evaluated the difference of *Salmonella* positivity status between two groups of farms, good practices adapted in broiler rearing at the project intervened farms, and non-project intervened traditional farms.

## Materials and Methods

### Ethical approval and informed consents

The farms were selected after consultation with the sub-district (Upazila) livestock office of each study site taking into consideration of willingness of the farmers. No ethical approval was required; however, during the collection of samples; verbal consent was taken from each of the farm owner/managers.

### Study area and study period

**Figure-1:**
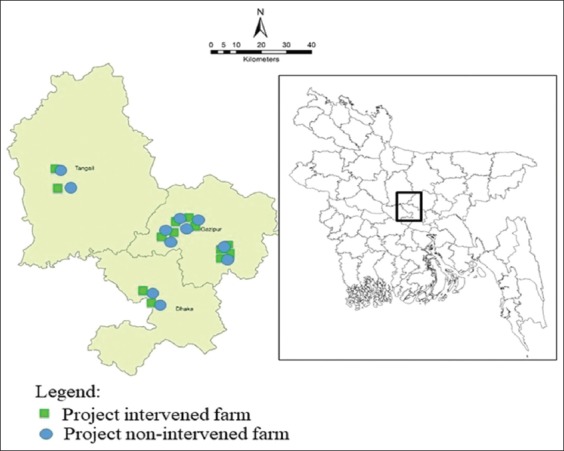
Location of the broiler farms, equal number farms (n=16) both in project intervened and non-intervened category were included from three districts of Bangladesh (as the coordinates of some farms are same, all farms are not visualized in the map).

The study was conducted in three different districts (Dhaka, Gazipur, and Tangail) of Bangladesh under this study from May 2017 to December 2017 (Figure-1). Dhaka district is located in between 23°22’30” and 24°22’20” north latitudes and in between 89°41’6” and 90°59’23” east longitudes. Gazipur district is located in between 23°53’ and 24°20’24” north latitudes and in between 90°04’ and 90°49’ east longitudes. Tangail district is located in between 23°59’50” and 24°48’51” north latitudes and in between 89°48’50” and 90°51’25” east longitudes.

### Farm selection, sample collection, and processing

Sixteen (16) project intervened farm with an inclusion criterion of a minimum flock size of ≥2000 that comprised 12 from Gazipur district, two from Dhaka, and two from Tangail district were included under this survey with good biosecurity and farm practices from May 2017 to December 2017. A similar number of farms (n=16) were included randomly from the same study sites to match the project intervened farm for comparing the best farm practices among the two groups.

Three hundred and fifty-two (352) different samples were randomly collected through convenient sampling technique from 32 broiler farms in three different districts, of which, 75% (n=264) samples (96 cloacal swab, 24 whole carcasses, 48 feed, 48 water, and 48 attendant hand rinse) were collected from 24 farms (n=12 project intervened, and n=12 project non-intervened) of Gazipur district, 12.5% (n=44) samples (16 cloacal swab, four whole carcasses, eight feed, eight water, and eight attendant hand rinse) were collected from four farms (n=2 project intervened, and n=2 project non-intervened) of Tangail district, and remaining 12.5% (n=44) samples (16 cloacal swab, four whole carcasses, eight feed, eight water, and eight attendant hand rinse) were collected from four farms (n=2 project intervened, and n=2 project non-intervened) of Dhaka district. Normal saline (0.85% NaCl) was used for the collection of cloacal swabs, 0.1% peptone water was used for the collection of attendants’ hand rinse water. After collection, samples were shifted to the Bacteriology and Molecular Microbiology Laboratory of the Department of Microbiology and Hygiene, Bangladesh Agricultural University maintaining proper cool chain using icebox. All collected samples were processed and cultures within 5-6 h of its collection time. The samples of 10 g of whole carcasses and 10 g of feed samples were performed homogenate using mortar and pestle and dissolved in 90 ml of 0.1% peptone water, respectively, for culture and further testing.

### Isolation and identification of *Salmonella* spp.

Isolation and identification of *Salmonella* spp. were carried out according to the methods described by Akbar and Anal and ISO 6579:2002(E) [3,23] with a little modification. Separately, the processed sample of 50 µl was taken and poured on Xylose Lysine Deoxycholate agar (XLD) (HiMedia, India) and spread using glass spreader, and then incubated at 37°C for 24 h. After incubation, cultural characteristics were observed, on XLD agar, *Salmonella* presented pink colonies having a black center. The suspected colonies were then subcultured on XLD agar and incubated again at 37°C for 24 h for obtaining pure colonies. For identification of suspected colonies, Gram’s stain, motility test, and different biochemical tests including sugar fermentation (dextrose, sucrose, lactose, maltose, and mannitol), methyl red, Voges–Proskauer, indole, citrate, and urease tests were accomplished. The isolated colonies were then subjected to molecular confirmation through polymerase chain reaction (PCR), antimicrobial susceptibility test, and serogrouping.

### Molecular detection of Salmonella spp.

For the molecular assay, the DNA template was equipped with the boiling method as described by Queipo-Ortuño *et al*. [24]. The 16S rRNA gene-based PCR was performed for the confirmation of the genus *Salmonella*. Primers were used for the amplification of the 16S rRNA gene according to the procedure described by Lin and Tsen [25] and shown in Table-1. The reaction mixture (20 µl) was prepared by mixing 10 µl master mixtures (Promega, USA), 1 µl forward primer (10 pmol), 1 µl reverse primer (10 pmol) (BioServe Biotechnologies Ltd., USA), 3 µl DNA template, and 5 µl deionized water. The PCR reactions were carried out using a thermocycler (Astec, Japan) with the following program: Initial denaturation with one cycle for 5 min at 94°C, 30 cycles each consist of denaturation with 30 s at 94°C, annealing with 30 s at 50°C, extension with 30 s at 72°C, and a final extension step of 5 min at 72°C. PCR products were analyzed by 2% agarose (Invitrogen, USA) gel electrophoresis and the bands were visualized with ultraviolet (UV) light after staining with ethidium bromide (0.5 µg/ml) for 10 min in a dark place. Bands were visualized and images were captured on a UV transilluminator (Biometra, Germany).

**Table-1:** The list of primers used for the identification of *Salmonella* spp.

Primer	Sequence (5’- 3’)	Target	Amplicon size (bp)	Reference
Sal 16S rRNA F	TGTTGTGGTTAATAACCGCA	*Salmonella* 16S rRNA gene	574	[25]
Sal 16S rRNA R	CACAAATCCATCTCTGGA			

### Serogrouping of Salmonella by O-antigen test

Serogrouping of isolated *Salmonella* spp. was done by slide agglutination test using commercial *Salmonella*-specific polyvalent O (A-I) antisera, *Salmonella* O Group B (Factor O: 4, 5, and 27) antisera, and *Salmonella* O Group D (Factor O: 9, 46) antisera kits (S & A Reagents Lab Ltd., Bangkok, Thailand) following the procedure described by Dhakal *et al*. [11].

### Antimicrobial susceptibility test

All isolated *Salmonella* spp. were confirmed on antimicrobial susceptibility test by disk diffusion method to determine antimicrobial profile following the method described by Bauer *et al*. [26] and Clinical and Laboratory Standards Institute (CLSI) [27]. The following eight commercially available antimicrobial disks (HiMedia, India) were used at indicated concentration (µg/disk): Amoxicillin (AMX, 30 µg), azithromycin (AZM, 30 µg), ciprofloxacin (CIP, 5 µg), erythromycin (E, 30 µg), gentamicin (GEN, 10 µg), norfloxacin (NOR, 10 µg), streptomycin (S, 10 µg), and tetracycline (TE, 30 µg) to determine the antimicrobial susceptibility patterns. After preparing 0.5 McFarland standards bacterial suspension using normal saline, a sterile cotton bud was dipped into the bacterial suspension. The excess fluid of a swab was removed by pressing firmly against the inside of the tube just above the fluid level. The bud was streaked over the entire surface of Mueller-Hinton agar (HiMedia, India) medium 3 times, rotating the plate approximately 60 degrees after each application to ensure an even distribution of the inoculums. The antimicrobial disks were placed individually using sterile forceps and then gently press down onto the agar. The plates were inverted and incubated at 37°C overnight. After incubation, the zone of growth inhibition (diameter) of each antimicrobial agent was measured according to the guidelines of CLSI [27].

### Data management and statistical analysis

The data were captured and recorded in Microsoft Excel^®^ worksheet and imported into Epi Info 7 program [28] for statistical analysis. A univariate logistic regression model was used to calculate the odds ratio (OR) for evaluating the association of best farm practices among two groups of farms (project-intervened and non-project-intervened) with p=0.05 were used to determine statistical significance. Proportion, percentage, and 95% confidence interval (CI) were calculated using an excel data analysis tool pack for estimating prevalence status in various parameters of two groups of farms.

## Results

### Prevalence estimation and isolation of Salmonella spp.

A total of 352 samples were collected from 32 broiler farms of three different districts where 50% (n=176) samples were collected from project intervened farms and rest 50% (n=176) samples were collected from non-project intervened farms. Of 352 samples, overall prevalence of *Salmonella* spp. was estimated at 31.25% (110/352) (95% CI=26.44%-36.38%), a prevalence of 24.43% (43/176) (95% CI=18.28-31.47) was estimated in the project intervened farms and 38.07% (67/176) (95% CI=30.87-45.68) prevalence in non-project intervened farms (Table-2).

**Table-2:** Prevalence of *Salmonella* spp. in broiler farms of three districts of Bangladesh (project intervened farms, n=176, and project non-intervened farms, n=176).

District	Category of farms	Number of sample (n)	Number of isolates (positive)	Prevalence	95% CI
Gazipur	Project intervened	132	33	25	17.88-33.28
	Non-project intervened	132	48	36.36	28.17-45.18
Overall Gazipur		264	81	30.68	25.17-36.62
Tangail	Project intervened	22	7	31.82	13.86-54.87
	Non-project intervened	22	9	40.91	20.71-63.65
Overall Tangail		44	16	36.36	22.41-52.23
Dhaka	Project intervened	22	3	13.64	2.91-34.91
	Non-project intervened	22	10	45.45	24.39-67.79
Overall Dhaka		44	13	29.55	16.76-45.20
Three districts (Gazipur, Tangail, and Dhaka)	Project intervened	176	43	24.43	18.28-31.47
	Non-project intervened	176	67	38.07	30.87-45.68
Overall (three districts)		352	110	31.25	26.44-36.38

CI=Confidence interval

**Figure-2:**
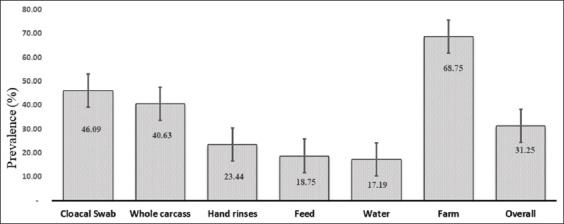
Frequency of prevalence of *Salmonella* spp. with a standard error of the mean at different parameters of broiler farming practices (farm=32, sample=352).

Of 128 cloacal swab samples, 46.09% (n=59) samples were found positive for *Salmonella* spp. (Figure-2). Similarly, of 64 feed samples, 64 water samples, 18.75% (n=12), and 17.19% (n=11) were found positive, respectively, for *Salmonella* spp. A total of 64 water samples, 64 farm attendant’s hand rinse water sample, 32 whole carcasses samples, 17.19% (n=11), 23.44% (n=15), and 40.63% (n=13), were shown positive for *Salmonella* spp. (Figure-2).

Of 32 farms, 68.75% (n=22) farms were found positive with *Salmonella* spp. of which 28.15% (n=9) farms were under project intervened category and remaining 40.63% (n=13) farms under non-project intervened category (Figure-2). The variation of prevalence among two groups of farms (project intervened and non-intervened) was observed among the three districts (Table-2). The non-intervened farms were found to be riskier than the project-intervened farms considering *Salmonella* positivity status (OR=1.9, 95% CI=1.20-3.00, p=0.005) and statistically found significant (Table-3).

**Table-3:** Univariable logistic regression analysis for associating the best farm practices between two groups of broiler farms with the likelihood of *Salmonella* infection in different parameters.

Parameter/sample type	Farm type	Positive	Negative	Odds ratio	95% CI	p value
Farm	Project non-intervened	13	3	3.37	0.71-16.06	0.12
	Project intervened	9	7			
Cloacal swab	Project non-intervened	34	30	1.84	0.91-3.72	0.08
	Project intervened	24	39			
Feed	Project non-intervened	4	24	1.17	0.26-5.17	0.83
	Project intervened	4	28			
Water	Project non-intervened	7	25	1.96	0.52-7.39	0.32
	Project intervened	4	28			
Attendants’ hand rinse water	Project non-intervened	22	10	0.41	0.12-1.34	0.14
	Project intervened	27	5			
Whole carcass	Project non-intervened	8	8	2.2	0.53-9.20	0.28
	Project intervened	5	11			
Overall	Project non-intervened	67	109	1.9	1.20-3.00	0.005
	Project intervened	43	133			

CI=Confidence interval

### Molecular detection by PCR

**Figure-3:**
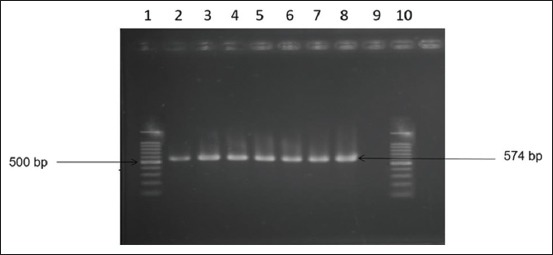
16S rRNA gene-based polymerase chain reaction of *Salmonella* spp. Lane 1 and 10: 100 bp DNA ladder; Lane 2-8: Tested samples were positive for the 16S rRNA gene, Lane 9: negative control without DNA.

Genus specific 16S rRNA gene-based PCR was performed for the confirmation of *Salmonella* isolates. All *Salmonella* isolates gave specific amplification (574 bp). The results of PCR are presented in Figure-3.

### Serogrouping of Salmonella spp.

Serogrouping of *Salmonella* isolates was performed by slide agglutination test using commercial *Salmonella* specific polyvalent O (A-I) antisera, *Salmonella* O Group B (Factor O: 4, 5, 27) antisera, and *Salmonella* O Group D (Factor O: 9, 46) antisera (S & A Reagent Lab). All isolates were positive to *Salmonella* Poly A-I antisera. Of 110 isolates, 39.1% (n=43) isolates were from project intervened farms, of which 30.23% (n=13) were classified under serogroup B (O:4,5,27) and 69.77% (n=30) under serogroup D (O:9,46). In other respects 60.9% (n=67) isolates were confirmed from non-project intervened farms, of which 32.84% (n=22) were classified under serogroup B (O:4,5,27) and 67.16% (n=45) under serogroup D(O:9,46). More than two-third (68.18%, 75/110) isolates of two categories of farms were classified under serogroup D (O:9,46) and rest of the isolates (31.82%, 35/110) were under the serogroup B (O:4,5,27)(Table-4).

**Table-4:** Summary of *Salmonella* spp. serogrouping.

Category	Isolates No.	Number (%) of *Salmonella* isolates		

		Poly A-I	Group B (O: 4,5,27)	Group D (O: 9,46)
Project intervened	43	100	13 (30.23)	30 (69.77)
Non-project intervened	67	100	22 (32.84)	45 (67.16)
Total	110	100	35 (31.82)	75 (68.18)

### Antimicrobial susceptibility of Salmonella spp.

**Figure-4:**
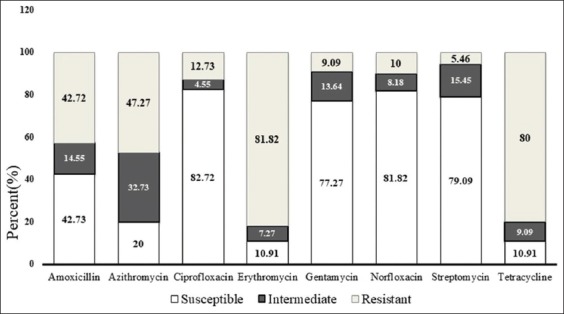
Proportion of antimicrobial susceptibility against eight selected antimicrobial agents is amoxicillin, azithromycin, ciprofloxacin, erythromycin, gentamycin, norfloxacin, streptomycin, and tetracycline presented in three categories (susceptible, intermediate, and resistant) of the pattern.

Antimicrobial susceptibility test was carried out in 110 *Salmonella* isolates against eight selected antimicrobial agents. The results of susceptibility analysis showed that 42.73%, 82.72%, 77.27%, 81.82%, and 79.09% of *Salmonella* isolates were susceptible to amoxicillin, ciprofloxacin, gentamycin, norfloxacin, and streptomycin, respectively. The resistance analysis showed that 42.73%, 47.27%, 81.82%, and 80% of *Salmonella* isolates were resistant to amoxicillin, azithromycin, erythromycin, and tetracycline, respectively (Figure-4).

### Antimicrobial resistance patterns of Salmonella spp.

The results of antimicrobial resistance patterns of *Salmonella* spp. are summarized in Table-5. Of 110 (n=110) *Salmonella* spp., 9.09% (n=10) isolates were resistant to one agent (TE), 6.63% (n=7) isolates were resistant to one agent (E), 3.64% (n=4) isolates were resistant to one agent (AMX), 1.82% (n=2) isolates were resistant against two agents (TE-AZM), 17.27% (n=19) isolates were resistant against three agents (TE-E-AMX), 30 27.27% (n=30) isolates were resistant against three agents (TE-E-AZM), 7.27% (n=8) isolates were resistant against three agents (TE-E-CIP), 4.55% (n=5) isolates were resistant against three agents (E-AMX-GEN), 14 12.73% (n=14) isolates were resistant against four agents (TE-E-AMX-AZM), 4.55 % (n=5) isolates were resistant against four agents (TE-AMX-GEN-NOR), and 5.45% (n=6) isolates were resistant against four agents (AMX-CIP-NOR-S) (Table-5).

**Table-5:** Antimicrobial resistance pattern of *Salmonella* spp.

Isolates	No. of agents	Antimicrobial resistance profile	No. (%) of isolates	No. (%) of multidrug resistant isolates
*Salmonella* spp.	No. resistance demonstrated	-	-	89 (80.91)
	1	TE	10 (9.09)	
	1	E	7 (6.63)	
	1	AMX	4 (3.64)	
	2	TE-AZM	2 (1.82)	
	3	TE-E-AMX	19 (17.27)	
	3	TE-E-AZM	30 (27.27)	
	3	TE-E-CIP	8 (7.27)	
	3	E-AMX-GEN	5 (4.55)	
	4	TE-E-AMX-AZM	14 (12.73)	
	4	TE-AMX-GEN-NOR	5 (4.55)	
	4	AMX-CIP-NOR-S	6 (5.45)	
	**Total resistant isolates**		**110 (100)**	

In this study, multidrug-resistant *Salmonella* spp. were identified and presented resistant against two or more antimicrobials, alarmingly, 80.91% (n=89) *Salmonella* isolates were established as multidrug-resistant in this survey (Table-5).

## Discussion

Despite the importance of the poultry sector in the National Economy of Bangladesh, insufficient disease data brings bottlenecks toward understanding the disease burden like its true prevalence, spatial and temporal distribution, and economic impact [29]. Among the different types of bacterial and viral origin diseases in Bangladesh, *Salmonella* infection is cogitated to be one of the major problems nowadays [9,10]. In view of that, this survey was rational to determine the status of *Salmonella* spp. in broiler farming system that will pave the way for a baseline data depository in the National Disease Control Program.

The study estimated the overall prevalence of *Salmonella* spp. was 31.25% (95% CI=26.44%-36.38%), in broiler farms of poultry dense districts of Bangladesh. The prevalence was found to be lower in project intervened farms (24.43%, 95% CI=18.28-31.47) than the non-project intervened traditional farm (38.07%, 95% CI=30.87-45.68). Due to intervention of good practices of biosecurity measures such as provision of perimeter fencing, netting of the farm, footwear clean entry in the farm, all-in all-out practice, and cleaning and sanitation practices, the likelihood of bacterial contamination was lessen in project intervened farms than the non-project intervened farms [30]. The project intervened farms were found to be protective considering *Salmonella* infection in this study (OR=1.9, 95% CI=1.20-3.00, p=0.005). This finding has validated the impact of good agriculture practices (GAP) on poultry rearing at the farmers’ level.

In this study, *Salmonella* spp. were isolated from hand rinses of farm attendants as the earliest research in Bangladesh. The presence of *Salmonella* in poultry attendants’ hand rinse water (23.44%, 15/64) could pose a serious impact on public health. This finding has partially dissenting with the studies of Paul *et al*. [7] and Akond *et al*. [31] as 50% and 6% poultry retailer hand rinse water were found positive, respectively. In this study, feed samples were found to be contaminated with *Salmonella* spp. (18.75%, 12/64). This finding was reasonably supported by several authors [32,33], as 28.56% *Salmonella* spp. described by Al-Mamun *et al*. [32]. A total of 64 water samples were tested, and 11 17.19% (n=11) samples were found positive. This finding is compatible with the findings of several researchers [32-34]. In this study, of 32 whole carcasses, 40.63% (n=13) samples were positive for *Salmonella* spp. This finding has similarities with the studies of a few investigators [14,32]; however, Karim *et al*. [8] showed only 20% *Salmonella* positivity in broiler meat. A total of 128 cloacal swab samples were collected, of which 46.09% (n=59) samples were found positive for *Salmonella* spp. This finding was in conformity with the studies by some researchers [14,31]; however, Paul *et al*. [7] showed a very high prevalence (80%) in the cloacal swab. The overall prevalence of *Salmonella* spp. in broiler farming system was 31.25% and the farm level prevalence was 68.75% (Figure-4). This finding was well-matched with the studies by several researchers [5,31,35]; nonetheless, Paul *et al*. [7] and Islam *et al*. [18] showed a relatively higher prevalence of *Salmonella* spp. from broiler farms as 53.33% and 66.67%, respectively.

In this study, isolation and identification of *Salmonella* spp. were done through culturing of sample on selective media, Gram’s stain, different biochemical tests, and finally confirmed by 16S rRNA gene-based PCR. This method also used by several researches [14,32,36-38].

Serogrouping of *Salmonella* isolates was performed by slide agglutination test using commercial *Salmonella*-specific polyvalent O (A-I) antisera, *Salmonella* O Group B (Factor O: 4, 5, 27) antisera, and *Salmonella* O Group D (Factor O: 9, 46) antisera. Among the 110 isolates, 31.82% (n=35) were under serogroup B and 68.18% (n=75) isolates were under to serogroup D. The most prevalent serogroup identified in this study was serogroup D. This conclusion was in agreement with the findings of Al-Mamun *et al*. [32]. About 30.56% (n=11) isolates were fitted to serogroup B and 69.44% (n=25) fitted to serogroup D.

The results of susceptibility test showed that isolated *Salmonella* spp. were highly resistant to erythromycin (81.72%) and tetracycline (80%), and moderately resistant to amoxicillin (42.73%) and azithromycin (47.27%). This finding is compatible with the studies by many researchers [5,14,31,32]; however, Islam *et al*. [18] showed that the isolated *Salmonella* spp. were highly sensitive and Ifeanyichukwu *et al*. [39] revealed as highly resistant to amoxicillin, and Akbar and Anal presented that the isolated *Salmonella* spp. from ready-to-eat poultry were highly susceptible to erythromycin [3]. On the other hand, most of the isolated *Salmonella* spp. were susceptible to ciprofloxacin (82.72%), gentamicin (77.27%), norfloxacin (81.82%), and streptomycin (79.09%) [7,14,18,31]. On the contrary, this finding is incompatible with the findings of several researchers, among them Ifeanyichukwu *et al*. [39] presented the data where isolated *Salmonella* spp. were found to be highly resistant to gentamycin, and Paul *et al*. [7] showed the data where ciprofloxacin was susceptible against merely 20% *Salmonella* isolates.

In this investigation, of 110 *Salmonella* isolates, 56.36% (n=62) and 22.73% (n=25) isolates showed resistance against at least three and four antimicrobial agents, respectively, and two or more antimicrobial agents 80.91% (n=89) isolates as multidrug-resistant. The latter finding is well-matched with the studies by several researchers [14,32]. The high proportion of multidrug resistance showed by the isolated *Salmonella* spp. may be the result of the unjudicial use of different types of antimicrobial agents in poultry production in Bangladesh with an aim to retard the bacterial infection [14]. In addition, some antibiotics are being used unscrupulously in poultry feed and water by the feed millers and farmers, respectively, and these are also be the another cause for showing multidrug resistance. The findings of this study suggested that multidrug-resistant *Salmonella* spp. isolated from broiler farms might be an important concern for public health.

## Conclusion

The presence of *Salmonella* spp. was confirmed from a wide range of samples at the broiler farming system specifies severe public health importance. This finding streamlines to GAP in poultry farming relating to maintaining proper farm biosecurity, all-in all-out practices, withdrawal period, safe disposal of poultry waste and prudent use of antimicrobial agents along with maintaining personal hygiene are needed to minimize the likelihood of *Salmonella* infection in poultry and its antimicrobial-resistant, and further transmission in human as a consequence. To minimize these public health threats, awareness creation and motivational activity on good hygienic practices and GAP for poultry farmers through participatory training under the “One Health” platform are very much necessitated.

## Authors’ Contributions

SMLK planned and designed the study. DM, MNU, BA, AHMTA, and SKSI assisted in data collection, laboratory work, data analysis, and drafting of the manuscript. SMLK, SKSI, MSI, and MSRK assisted in data analysis and drafting of the manuscript. All authors read and approved the final manuscript.
